# MMP-2 Silencing through siRNA Loaded Positively-Charged Nanoparticles (AcPEI-NPs) Counteracts Chondrocyte De-Differentiation

**DOI:** 10.3390/polym15051172

**Published:** 2023-02-25

**Authors:** Raffaele Conte, Mauro Finicelli, Alessia Borrone, Sabrina Margarucci, Gianfranco Peluso, Anna Calarco, Michela Bosetti

**Affiliations:** 1 Elleva Pharma s.r.l., via P. Castellino 111, 80131 Naples, Italy; 2 Research Institute on Terrestrial Ecosystems (IRET)—CNR, Via P. Castellino 111, 80131 Naples, Italy; 3 Dipartimento di Scienze del Farmaco (DSF), Università Piemonte Orientale “A. Avogadro”, Largo Donegani 2, 28100 Novara, Italy; 4 Faculty of Medicine and Surgery, Saint Camillus International University of Health and Medical Sciences, Via di Sant’Alessandro 8, 00131 Rome, Italy

**Keywords:** nanoparticles, acetylated-PEI, siRNA, MMPs, chondrocytes, de-differentiation

## Abstract

The abnormal matrix remodeling process, as well as inflammation, angiogenesis, and tumor metastasis, are related to an increase in the synthesis and secretion of matrix metalloproteinases (MMPs), the zinc-dependent proteolytic endopeptidases. Recent studies have evidenced MMPs’ role in osteoarthritis (OA) development, during which chondrocytes undergo hypertrophic differentiation and exhibit enhanced catabolism. The trait of OA is extracellular matrix (ECM) progressive degradation regulated by many factors, in which MMPs play an important role, which indicates them as potential therapeutic targets. Herein, a small interfering RNA (siRNA) delivery system able to suppress MMPs’ activity was synthetized. Results demonstrated that positively charged nanoparticles (AcPEI-NPs) complexed with MMP-2 siRNA are efficiently internalized by cells with endosomal escape. Moreover, avoiding lysosome degradation, MMP2/AcPEI nanocomplex increases nucleic acid delivery efficiency. Gel zymography, RT-PCR, and ELISA analyses confirmed MMP2/AcPEI nanocomplex activity even when embedded within collagen matrix resembling the natural extracellular matrix. Further, the inhibition of in vitro collagen degradation exerts a protective effect on chondrocyte dedifferentiation. The suppression of MMP-2 activity, preventing matrix degradation, protects chondrocytes against degeneration and supporting ECM homeostasis in articular cartilage. These encouraging results promote further investigation to validate the utilization of MMP-2 siRNA as ‘‘molecular switch’’ able to counteract osteoarthritis.

## 1. Introduction

Osteoarthritis (OA) is a degenerative joint disease prevalently characterized by an inflammatory environment, articular cartilage damage, and bone shape change. Symptoms are related to pain, stiffness, and loss of mobility that affect patients’ quality of life and produce an economic burden both on individuals and society [1,2,3]. Due to the multifactorial OA etiology, there are no effective pharmacological treatments. Indeed, official recommendations aim at controlling symptoms until the severity of the disease mandates surgical intervention with joint replacement [4]. In addition, non-steroidal anti-inflammatory drugs has critical limitations such as low bioavailability, a short half-life, poor targeting, and high systemic toxicity [5,6]. For instance, gene therapy could be used to modify the expression of the endogenous genes to prevent or slow down the OA’s pathological progress [7]. Compared with the naked genetic molecules, polymeric nanoparticles hold the potential to provide safe, efficient, and controllable gene delivery by manipulating properties such as surface charge, stability, endocytosis, endosomal escape, and toxicity [8,9]. Synthetic biodegradable polymers, such as Poly lactic-co-glycolic acid (PLGA), have several characteristics that make them suitable for non-viral delivery systems, including biocompatibility, low immunogenicity, and improved stability in biological fluids) [10]. Moreover, PLGA belongs to a family of FDA-approved biodegradable polymers. Indeed, PLGA hydrolysis leads to two endogenous monomers: lactic acid and glycolic acid, which are readily metabolized by cells via the Krebs cycle [11]. PLGA’s tunable properties enable surface functionalization with cationic molecules such as polyethyleneimine (PEI). PEIs form non-covalent complexes with nucleic acids, thus protecting siRNAs from degradation, mediating cellular uptake, and efficiently promoting lysosomal protection and escape into the cytoplasm. Although PEI-derived nanoparticles with highly positive surface charges have been shown to be effective vehicles for the delivery of siRNA and miRNA, non-specific interaction with the negatively charged serum proteins leads to aggregate formation and rapid clearance from the intended treatment area by the reticuloendothelial system [12]. In addition, the high charge density due to PEI can also contribute to damage to the cell membrane and subsequent cell death [13]. In a previous work, Calarco et al. demonstrated that acetylation of primary and secondary amines on branched PEI (AcPEI) alters the protonation behavior of the cationic polymer, leading to a reduction of both the cytotoxicity and the genotoxicity of PEI and an enhancement of gene delivery activity [14].

In the present work, acetylated nanoparticles (AcPEI-NPs) have been used as non-viral gene delivery to inhibit matrix metalloprotease 2 (MMP-2) proteolytic activity. Recently, MMPs, especially MMP-1, MMP-2, and MMP-9, have emerged as the leading candidates for involvement in the OA disease progression [15,16]. Indeed, the progressive degradation of the articular cartilage is sustained by increased activity of matrix-degrading metalloproteinases. Moreover, during the process of OA, chondrocytes undergo hypertrophic differentiation and exhibit enhanced catabolism [17], in which MMPs are particularly involved [18]. In this context, a gene delivery system able to interfere with MMPs’ activity may become an interesting intervention to stop or slow down the OA degenerative process. To this aim, AcPEI-NPs complexed with MMP-2 siRNA (MMP2/AcPEIx) were synthesized, and their ability to limit matrix degradation was evaluated by a 3D in vitro model that mimicked the catabolic microenvironment typical of OA disease. Gel zymography, RT-PCR, and ELISA analyses demonstrated the ability of MMP2/AcPEI nanocomplex embedded within collagen matrix to inhibit in vitro collagen degradation exerting a protective effect on chondrocyte dedifferentiation. These encouraging results promote further investigation to validate the utilization of MMP-2 siRNA as ‘‘molecular switch’’ able to counteract osteoarthritis.

## 2. Materials and Methods

### 2.1. Cell Culture and Materials

d,l-Lactide/glycolide copolymer (PLGA, PURASORB^®^, inherent viscosity 0.20 dL/g) was a generous gift from PURAC (Gorinchem, The Netherlands). Branched polyethylenimine (PEI, MW: 25 kDa), poly(vinylalcohol) (PVA), acetic anhydride, 5,5-dimethyl-pyrroline N-oxide (DMPO), phenyl N-tert-butylnitrone (POBN), 1-Ethyl-3-(3-dimethylaminopropyl)-carbodiimide (EDC), N-hydroxysuccinimide (NHS), 2,4,6-trinitrobenzenesulfonic acid (TNBS), D_2_O, etoposide, dimethyl sulfoxide (DMSO) were obtained from Sigma Aldrich (Milan, Italy). All the other chemicals and reagents used were of analytical purity grade or higher. MMP-2 siRNA, scramble siRNA (sh) and Tris-EDTA (TE) buffer were obtained from Thermo-Fisher Scientific (Milan, Italy), and MISSION^®^ siRNA (siRNA) Fluorescent Universal Negative Control was obtained from Sigma-Aldrich. Human prostate cancer cell lines (PC3, ECACC, UK) were cultured in Dulbecco’s Modified Eagle Medium DMEM:F12 medium (Sigma Aldrich) supplemented with 10% fetal bovine serum (FBS, Sigma Aldrich), 50 units/mL penicillin, and 50 mg/mL streptomycin. All cells were cultured in a humidified, 5% CO_2_ atmosphere tissue culture incubator at 37 °C. Human chondrocyte cells line C20A4 were obtained from the American Type Culture Collection (ATCC, Manassas, VA, USA) and maintained at 37 °C in a humidified atmosphere containing 5% CO_2_ in DMEM:F12 supplemented with 10% FBS, 1% L-glutamine, 50 U/mL penicillin, 50 mg/mL streptomycin, 50 μg/mL ascorbic acid, and 50 μM α-tocopherol (Euroclone, Milan, Italy). All cells were tested for contamination, including *Mycoplasma*, and used within 2 to 4 months. All experiments were performed with an 80% confluent monolayer.

For 3D collagen cultures, an injectable gel was obtained from rat tail tendons according to [19]. Briefly, rat tail tendons were sterilized by UV radiation for 48 h, extracted in 0.5 M acetic acid at 4 °C for 2 days, and the gross insoluble materials were removed by passing the solution through mesh. The final solution contains 2 mg/mL of soluble collagen as estimated from BCA (BCA Protein Assay kit, Pierce, Milan, Italy) and Sirius Red assay according to ref. [20].

### 2.2. Zymography

MMP-9 and MMP-2 levels in culture media were examined by gelatin zymography after 24 and 48 h of PC3 culture. The samples were diluted in non-reducing sample buffer (250 mM Tris–HCl pH 6.8, 30% glycerol, 6% SDS, and 0.05% bromophenol blue) with a 3× volume of culture media and then subjected to electrophoresis on a 10% SDS-PAGE gel co-polymerized with 2 mg/mL gelatin. Following electrophoresis at 100 V for approximately 2.5 h, gels were rinsed in 2.5% Triton X-100 for 1.5 h at room temperature (25 °C) and incubated in an enzymatic activation buffer (50 mM Tris-HCl, 150 mM NaCl, 50 mM CaCl_2_, pH 7.6) for 18 h at 37 °C with gentle shaking. Gels were subsequently stained with 0.2% Coomassie Brilliant Blue R-250, 40% methanol, and 10% acetic acid for 20 min, and then de-stained in 5% methanol and 7% acetic acid for 1 h. A commercially available zymography marker (Gelatin Zymo MMP Marker; Life Laboratory, Yamagata, Japan) including human MMP-9 (MW: 92 or 82 kDa) and MMP-2 (72, 68, or 62 kDa) was run on each gel as a positive control. MMP levels were assessed based on gelatinolytic activity, indicated as clear bands against the dark blue background, and revealed with the VersaDoc instrument (Bio-Rad, Milan, Italy). Acquired pictures were quantitatively evaluated using the ImageJ program (NIH program), comparing the image assessment value of each unknown band to the value of the MMP standard band. The ratio of unknown to standard protein was calculated, and an arbitrary unit (au) value was assigned to each sample. Experiments were run in triplicate.

### 2.3. Nanocomplexes Preparation and Characterization

Acetylated nanoparticles (AcPEI-NPs) were prepared and characterized as reported in Calarco et al. [14]. Nanoparticles were complexed with MMP-2 siRNA (MMP2/AcPEI) or scramble-siRNA (sh/AcPEI) at nitrogen to phosphate (N/P) ratios ranging from 1 to 15 simply by incubating siRNA solution (300 pM) with the desired amount of AcPEI nanoparticles in RNAse-free TE buffer for 30 min at room temperature. Fluorescent nanocomplexes were obtained by incubating AcPEI-NPs with MISSION^®^ siRNA Fluorescent Universal Negative Control (siRNA/AcPEI). Dynamic light scattering (DLS) with a Malvern Zetasizer (Malvern Instruments Ltd., Malvern, UK) was used to measure the diameter and zeta potential of the nanocomplexes as reported by Conte et al. [21]

### 2.4. Nanocomplexes Uptake in 2D and 3D in Vitro Model

The capability of nanocomplexes to be internalized by cells was studied in standard culture conditions (2D cultures) and in 3D collagen gels (3D). For quantitative determination of nanocomplex internalization in 2D, cells were incubated for 1 h with different concentrations (10–300 µg/mL) of siRNA/AcPEI or with a fixed concentration of nanocomplexes (300 µg/mL) for a time-dependent uptake (5 min to 1 h). Before analysis, cells were extensively washed with PBS and Trypan Blue (0.4%) to quench the fluorescence of membrane-associated nanoparticles. Cell fluorescence was quantified by a laser scanning cytometer, FACS Calibur (Beckton Dickinson, NJ, USA). A total of 5000 cell events were analysed. For nanocomplexes-loading gel, siRNA/AcPEI (150 µg/mL, 15:1) were added to the collagen solution prior to gel formation. Fluorescent siRNA released in PBS for 15 days was quantified using a plate reader (ASHI, Milan, Italy) at 480 nm/520 nm. For 3D cultures, cells were suspended in a culture medium and seeded at a density of 2 × 10^5^ cells/mL in nanocomplexes-loading gel prior to gel formation allowed to set at 37 °C for 1 h before addition of culture medium. Furthermore, gel was washed in PBS to remove surface-associated siRNA/AcPEI and incubated at 37 °C/5%CO_2_ for 15 days in culture medium. Culture media was changed twice a week.

### 2.5. Intracellular Localization of Nanoparticles

After 1 h of time lapse microscopy, cells were fixed for 30 min with 4% formaldehyde solution in PBS at room temperature, then washed with PBS and stained with the antibodies for RAB5 and LAMP1, to evidence endosomal and/or lysosomal colocalization. Briefly, cells were incubated O/N at 4 °C with primary antibodies: LAMP1 (20 μg/mL) and RAB5 (2 μg/mL) (Abcam, Cambridge, UK). After PBS washing, cells were incubated for 1 h at room temperature with Texas Red anti-mouse (Vector Lab, CA, USA) and the IRIS 5-goat anti-mouse (Cyanine Technologies, Torino, Italy) secondary antibodies, respectively. Dried cells were mounted with an anti-photobleaching medium (Vector) and observed at confocal microscopy (Leica DM IRE2) at 40× magnification. Images were acquired after excitation with an Argon laser at 488 nm, a Helium-Neon-Green laser excitation at 543 nm, and a Helium-Neon-Red laser excitation at 633 nm. siRNA/AcPEI were shown as green spots at 510 nm emission, Texas RED positive RAB at 620 nm emission, and IRIS positive LAMP at 680 nm emission. Images were recorded separately in each fluorescence channel and merged afterwards.

### 2.6. Silencing Efficiency of MMP2/AcPEI in 2D and 3D Culture

For MMP-2 silencing experiments, PC3 cells were seeded both in 2D and 3D cultures and treaded for 72 h or 15 days, respectively, as reported in Section 2.3. Afterward, 2D cultured cells were washed twice with PBS, and RNA was extracted using QIAzol reagent (Qiagen, Milan, Italy) according to the manufacturer’s instructions and retro-transcribed as described in De Luca et al. [10]. Quantitative Real-Time PCR (qRT-PCR) was performed as reported in Section 2.6. For 3D culture, collagen gels were harvested and homogenized in QIAzol reagent, and qRT-PCR was conducted as above reported.

The ability of MMP2-AcPEI to interfere with collagen degradation was analyzed for 24 days in the presence of transfected PC3 cells entrapped in the 3D gel (MMP2-3D). A cell-free collagen scaffold was used as negative degradation control (CTR-), while a cell-free collagen scaffold with 10 µL of collagenase type I (0.5 mg/mL, Sigma Aldrich) was considered as positive degradation control (CTR+). At each time point (0, 6, 12, 18, and 21 days), collagen scaffolds were rinsed in PBS and fixed overnight in 10% buffered formalin (Diapath S.r.l., Martinengo, Italy) at 4 °C before being stained with Sirius red F3BA (1 mg/mL, Chroma, Stuttgart, Germany) in picric acid (Sigma Aldrich). After 2 h, gels were extensively washed with acidic water and then with tap water. Measurements were completed using an inverted microscope equipped with a digital camera connected to an image analysis system (all from Leica Microsystems).

### 2.7. RNA Isolation, Reverse Transcription, and Quantitative Real-Time PCR (qRT-PCR)

Chondrocytes embedded in the collagen matrix were incubated for 7 or 21 days with conditioned medium of MMP2-AcPEI transfected PC3 or conditioned medium of sh-AcPEI transfected PC3. At each time point, cells were washed twice with PBS, and total RNA was extracted using QIAzol reagent (Qiagen, Milan, Italy) according to the manufacturer’s instructions and retro-transcribed as previously described [11]. For retro-transcription, total RNA (0.5 μg) was treated as described in EuroClone standard protocol and amplified by qPCR. Specific primers for Matrix Metalloproteases-2 (*MMP-2*), Collagen Type I Alpha 1 Chain (*COL1A1*), Collagen Type II Alpha 1 Chain (*COL2A1*), Aggrecan (*ACAN*), and β-Actin (*ACTB*) were used and listed in Table 1. qRT-PCR was run on a 7900 HT fast real-time PCR System (Applied Biosystem). The reactions were performed according to the manufacturer’s instructions using SYBR Green PCR Master Mix (Euroclone). All reactions were run in triplicate, normalized to the housekeeping gene (ACTB), and the results expressed as mean ± SD. The 2^−ΔΔCt^ method was used to determine the relative quantification.

The ratio of *COL2A1*/*COL1A1* was expressed as 2^_(CpCOL2A_CpGAPDH)/2^(CpCOL1A_CpGAPDH).

### 2.8. Western Blotting

Polyacrylamide gel electrophoresis was carried out in triplicate, according to Valentino et al. [22]. Membranes were probed with an MMP2 primary antibody (Cell Signaling Technology, Milan, Italy), followed by a secondary antibody conjugated with horseradish peroxidase. The bands were quantified densitometrically using Quantity One 1-D analysis software (BioRad, Milan, Italy).

### 2.9. Statistical Analysis

Data were expressed as means ± SD of at least three independent experiments. Statistical comparisons between the different experimental groups and their corresponding controls were made with a Student’s *t*-test, accepting *p* < 0.05 as the level of significance, using GraphPad Prism 6 software (GraphPad Software Inc., San Diego, CA, USA).

## 3. Results and Discussion

### 3.1. PC3 Cells Were Able to Degrade Collagen Matrix

**Figure 1:**
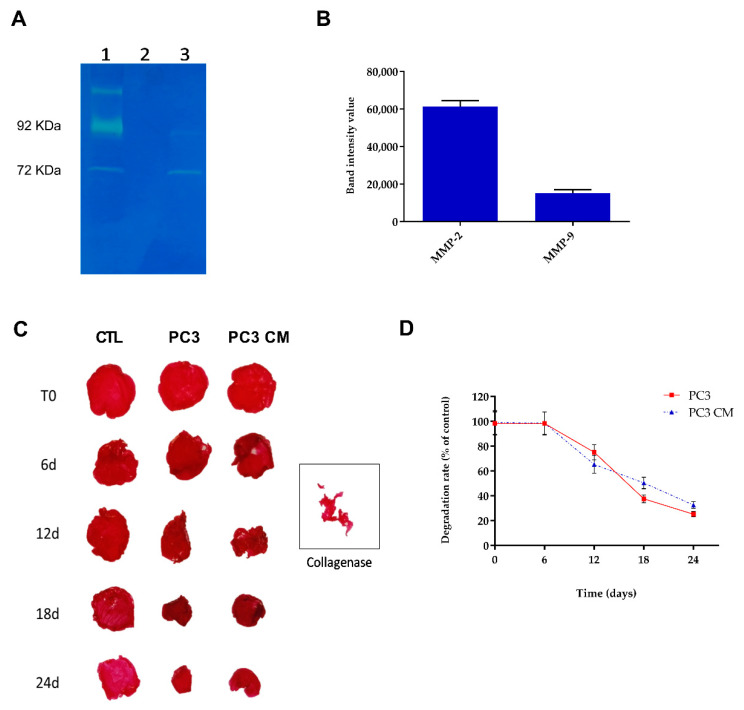
(**A**) Gelatinase zymogram of PC3 cells conditioned medium after 48 h of culture corresponding to the molecular weight of MMP-2 (72 kDa) and MMP-9 (92 kDa). (1-Markers, 2-empty lane, 3-PC3). (**B**) Quantitative densitometric analysis of MMPs expression of PC3 conditioned medium gelatinase zymogram. Data represented as mean ± SEM of three independent experiments. (**C**) Representative photomicrographs of collagen matrices stained with Sirius Red dye after 0, 6, 12, 18, 24 days in presence of PC3’s culture medium (PC3 CM) and in scaffolds containing PC3 cells (PC3). Collagenase was used as positive control. (**D**) Quantitative data of collagen degradation expressed as percent respect to control. Data represented as mean ± SEM of three independent experiments.

To mimic the catabolic microenvironment typical of OA disease, an in vitro 3D system was developed using a prostate cancer cell line (PC3) [23]. Under basal conditions, cultured human prostate cancer cells (PC3) expressed pro–MMP-2, which was released into the cell culture medium. As reported in Figure 1A, PC3 cells are able to secrete elevated quantities of MMP-2 and MMP-9 after 48 h of culture. Gelatinase zymography displayed a weak band for MMP-9 (92 kDa) and one band corresponding to the molecular-weight of MMP-2 (72 kDa). Moreover, the ability of MMPs released from PC3 cells to degrade collagen matrix was evaluated both in cell-free scaffolds cultured in the presence of PC3’s conditioned medium (PC3 CM) and in scaffolds containing PC3 cells (PC3) after 0, 6, 12, 18, and 24 days (Figure 1C,D). Macroscopic appearance and morphometric measurements were compared with those of the negative control (collagen in the presence of culture medium) and the positive control (collagen cultured in the presence of collagenase). Collagen scaffold degradation occurs after 12 days of culture in both PC3 and PC3 CM matrices with respect to the control. Moreover, no visual difference was detectable in the control at all time points tested (Figure 1C).

The ECM of healthy and arthritic joints is constantly subjected to remodeling processes [24]. In normal conditions, cartilage tissue homeostasis is achieved by the balance between MMPs and their tissue inhibitors (TIMPs). An imbalance of MMP/TIMP regulation, however, results in tissue destruction and functional alteration or local inflammation, leading to several diseases, including osteoarthritis (OA) [25]. Tissue inhibitors of matrix metalloproteinases have been shown to be elevated in patients with OA, but it is not clear if they are elevated as a compensatory response to the increased MMPs or if they are in some way contributing to the progression of the disease [26]. MMPs are normally expressed at low levels in the normal joint; however, their expression levels and activation states can change dramatically during the progression of OA [27]. Among the different MMPs, the family of gelatinases (MMP-2/gelatinase A and MMP-9/gelatinase B) play a primary role in the catabolism of articular collagen scaffolds due to their ability to degrade aggrecan and extracellular matrix proteoglycan, as well as collagen types I, II, and III, resulting in irreversible destruction within the joint [28]. Despite significant levels of MMP-2 and -9 being found at the site of cartilage destruction and in synovial fluid samples from OA patients, there is no effective treatment due to the low selectivity and side effects of MMP-2 and -9 chemical inhibitors [29].

### 3.2. AcPEI Nanocomplexes Are Efficiently Uptaken by Cells

Having previously proven the efficacy and tolerability of AcPEI, in this work its application is extended to achieve an efficient MMP-2 knockdown in PC3 cells used as model of degrading matrix. AcPEI nanoparticles were obtained following the well-established nanoprecipitation method as reported by Conte et al. According to our previous work, the hydrodynamic diameter of synthesized nanoparticles was around 108.6 ± 2.8 nm with a polydispersity index of 0.2 and a zeta potential of 21.6 ± 1.5 mV [21].

**Figure 2:**
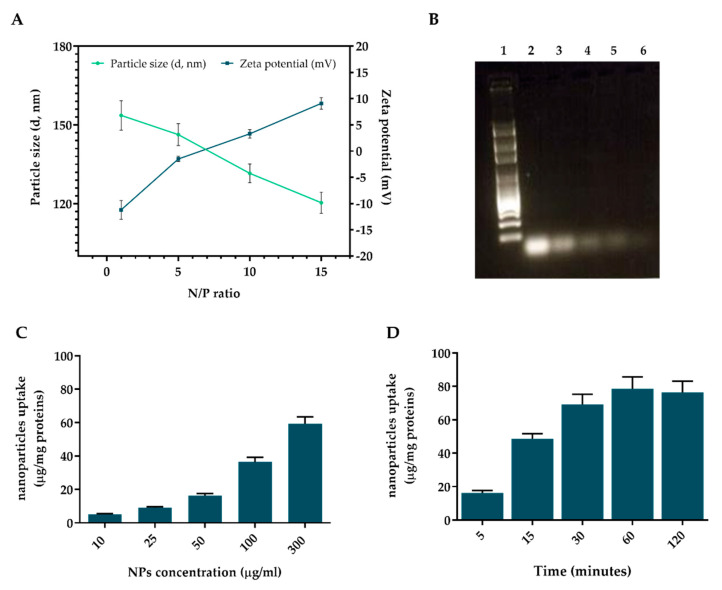
Characterization of siRNA/AcPEI complexes. (**A**) Average hydrodynamic diameter and zeta potential of AcPEI NPs at different N/P ratios (mean ± SD, n = 6). (**B**) Nuclease stability of siRNA/AcPEI complexes at different N/P ratios after incubation at 37 °C and 5% CO_2_ in 90% FBS. After gel electrophoresis, band intensities were quantified with ImageJ software and plotted versus time. Free MMP2-siRNA was used as control. Line 1: marker, line 2: free siRNA, line 3: N/P 1:1, line 4: N/P 5:1, line 5: N/P 10:1, and line 6: N/P 15:1. Uptake efficiency of fluorescent nanocomplexes (siRNA/AcPEI) in human prostate cancer cells PC3 at N/P ratio of 15:1. (**C**) Concentration and (**D**) time dependent cellular uptake of siRNA/AcPEI performed by FACS analysis. Cell-associated fluorescence was calculated from the standard curve and expressed as the number of nanoparticles (μg) uptaken per mg cell protein. Results are expressed as the mean of three independent experiments ± standard deviation (n = 3).

AcPEI-siRNA nanocomplexes were prepared by adding MMP-2-siRNA to different concentrations of AcPEI-NPs solution (MMP2/AcPEI_x_). As shown in Figure 2A, nanocomplexes size decreases with the increase in the N/P ratios from 1:1 to 15:1. In addition, siRNA complexes exhibit a negative zeta potential at low N/P ratios due to the negatively charged siRNA in the complex (Figure 2A). As the N/P ratio increases, the zeta potential of the nanocomplex shifts from negative to positive due to the neutralization of the siRNA by PEI. Particle size distributions and zeta potential curves of MMP2/AcPEIx are shown in Figure S1A,B. The ability of AcPEI-NPs to address siRNA protection from nuclease degradation was determined in FBS (Figure 2B). With increasing N/P ratios (from 1:1 to 15:1), the amount of intact MMP2-siRNA detected in the supernatant increased in parallel from 10% (N/P = 1:1) to >85% (N/P = 15:1) after 3 h of incubation. As the incubation time increases, the amount of intact siRNA decreases at a N/P ratio lower than 10:1, while free siRNA, used as a control, is already completely degraded after 1 h of incubation. Cell internalization is the first prerequisite of a nucleic acid delivery system. Therefore, to determine nanocomplex uptake efficiency, a fluorescent siRNA (MISSION^®^ siRNA, siRNA/AcPEI) was used. Flow cytometry analysis (FACS) of treated PC3 indicated that the kinetics of internalization are concentration-dependent in the range of 10–300 µg/mL (Figure 2C) with considerable nanocomplex internalization within 15 min (48.6 ± 3.1%) at a constant particle mass (150μg/mL), reaching a saturation point after 60 min (78.6 ± 6.3%, Figure 2D).

Based on data from experiments, an N/P ratio of 15:1 (MMP2/AcPEI_15_) was selected for in vitro silencing studies.

### 3.3. AcPEI Nanocomplexes Are Able to Endosomal Escape

Typically, nanoparticles are internalized into mammalian cells via endocytosis due to their size being too large to diffuse across cellular membranes [30]. However, entrapment of the delivery system in endocytic vesicles (endosomes and lysosomes) results in poor bioavailability of cargo, particularly for RNA or proteins [31,32]. Indeed, the low lysosomal pH (5.5–3.5) as well as the strong endosomal enzymatic activity might lead to drug degradation. Moreover, the therapeutic efficiency can be further decreased by the fast exocytosis of the nanocarriers [33]. PEI was able to overcome the “bottleneck” of endocytic vesicles due to the so-called “proton sponge effect” [34]. Most specifically, thanks to the protonation of tertiary amines, PEI exhibits high buffering capability at low pH, promoting an influx of protons inside the acidic cellular compartments via ATPase proton pumps and the consequent rupture of the organelle membrane due to an osmotic imbalance. The proton sponge effect of PEI is a generally accepted hypothesis in literature; however, it is important to mention that this concept is still heavily debated.

**Figure 3:**
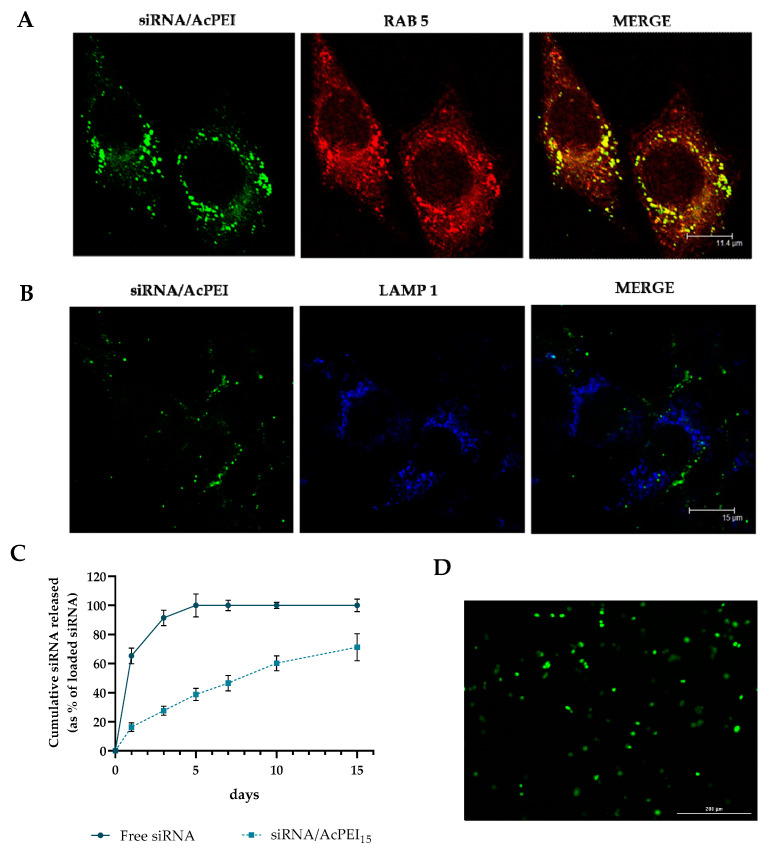
(**A**) Representative confocal microscopy images showing Cy5-siRNA colocalization with early endosomes tagged via a Rab5-Texas red fusion protein (scale bars, 15 µm). Yellow dots indicate regions of siRNA colocalized with Rab5 early endosomes. (**B**) Representative confocal microscopy images showing Cy5-siRNA colocalization with lysosomes tagged via a LAMP1. Areas of colocalization appear yellow in the merged image (scale bars, 200 µm). (**C**) Cumulative release profile of free siRNA and siRNA/AcPEI loaded in collagen matrix. The bars represent the means ± standard deviation (n = 3). (**D**) Confocal microscopy of PC3 cells cultured in 3D collagen matrix loaded with fluorescent nanocomplexes. Fluorescence micrograph at 20× magnification.

Colocalization studies with early endosomes were demonstrated with anti-RAB5 antibody with an immuno-fluorescence assay in confocal microscopy (Figure 3A), but colocalization with the lysosome marker LAMP1 was absent (Figure 3B). Considering that the final target destination of gene/siRNA delivery is, respectively, nuclear and cytoplasm targeting, endosomes escape is successfully reached by our system. This result is an important conclusive step, which suggests the conserved ability of AcPEI to activate at intracellular level the proton sponge effect. In addition, siRNA complexed with nanoparticles was retained in the hydrogel with a small burst of about 20% in the first three days, followed by a slowly released rate reaching 53.11 ± 5.18% over 7 days and 71.21 ± 9.35 at 15 days. On the contrary, free siRNA was released almost completely after 3 days (Figure 3C). Figure 3D shows a representative image of PC3 cells after 15 days of contact with siRNA/AcPEI in the 3D collagen matrix.

These data suggested that AcPEI nanoparticles were efficient in protecting siRNA from lysosome degradation, increasing nucleic acid delivery efficiency.

### 3.4. MMP2/AcPEI Inhibits MMP-2 Expression and Activity in PC3 Cells

Under OA conditions, chondrocytes up-regulate the production of proteases, including matrix metalloproteinases (MMPs) and aggrecanases. These proteases degrade the cartilage matrix, releasing matrix degradation products and protein fragments. These fragments then initiate further inflammatory responses, leading to a vicious cycle of cytokine production and cartilage destruction. Additionally, inflammatory cytokines and alarmins act on the adjacent synovium and bone to stimulate synovial inflammation and de-regulate peri-articular bone remodeling [35,36].

**Figure 4:**
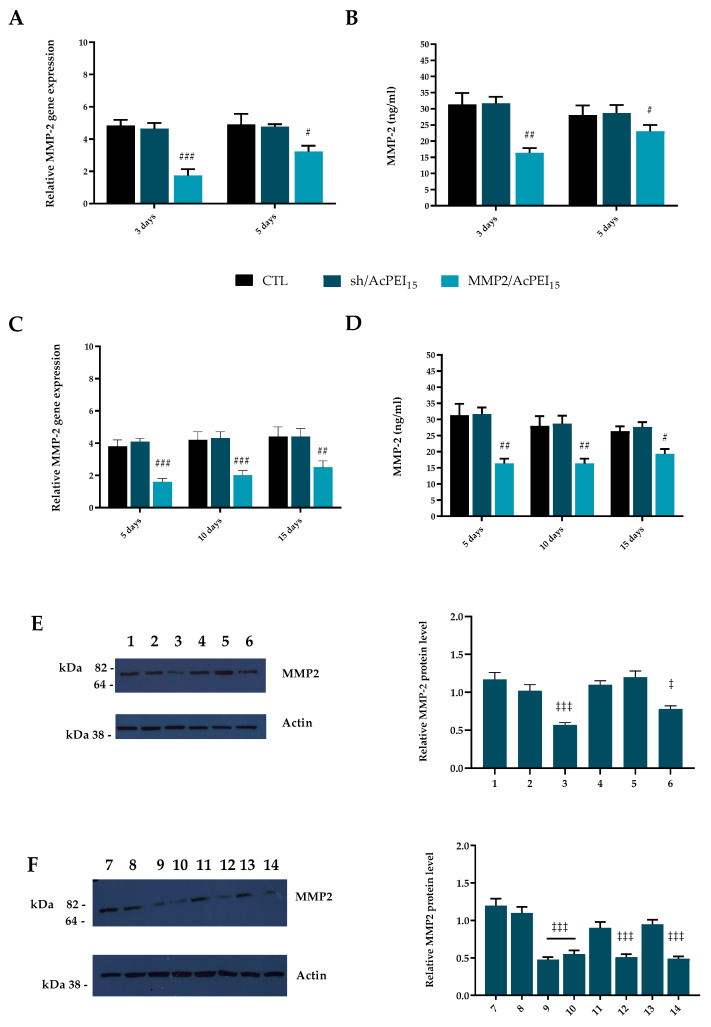
(**A**,**C**) Quantitative RT-PCR analysis of matrix metalloproteinase 2 (*MMP-2*) gene expression in PC3 cells cultured for 3, and 5 days in presence of MMP2/AcPEI_15_ or sh/AcPEI_15_. Non-transfected cells are used as control (CTL). The target gene expression was normalized to the housekeeping genes *HPRT1* and *GAPDH*. Relative differences in the PCR results were calculated using the comparative CT (2^−ΔΔCt^) method. (**B**) ELISA assay of MMP-2 in conditioned medium of PC3 treated with MMP2/AcPEI_15_ or sh/AcPEI_15_ for 3 and 5 days and in (**D**) for 5, 10 and 15 days. Non-transfected cells are used as control (CTL). (**E**,**F**) Western blot analysis of MMP2 protein was performed on total C20A4 protein fraction. (**E**) (1: CTL, 2: sh/AcPEI_15,_ 3: MMP2/AcPEI_15)_) 3 days; (4: CTL, 5: sh/AcPEI_15_, 6: MMP2/AcPEI_15_) 5 days. (**F**) 7: CTL, 8: sh/AcPEI_15_, 9: MMP2/AcPEI_15_ 3 days, 10: MMP2/AcPEI_15_ 5 days, 11: sh/AcPEI_15_, 12: MMP2/AcPEI_15_ 10 days, 13: sh/AcPEI_15_, 14: MMP2/AcPEI_15_ 15 days. The protein expression was normalized to the housekeeping protein Actin. The bars represent the means ± standard deviation (n = 3). # *p* < 0.05, ## *p* < 0.01 and ### *p <* 0.001 versus CTL and sh/AcPEI_15_.

The regulation of MMPs production by cartilage is complemented at the genetic level, as most of the MMPs genes are only expressed when tissue remodeling occurs. Among the MMPs, four (MMP-1, MMP-2, MMP-13, and MMP-14) are systematically expressed in adult cartilage, participating in tissue metabolism and increasing only in abnormal conditions [37,38]. In particular, the catabolic activity of MMP-2 is strongly regulated at the transcriptional and post-transcriptional levels [35]. shRNA (sh/AcPEI_15_) was used as the control. At 3 days after in vitro transfection, qRT-PCR analysis (Figure 4A) demonstrated a significant reduction of MMP-2 mRNA levels in the MMP2-treated group compared with those in the sh/AcPEI_15_-treated group and non-transfected cells (*p* < 0.05), corresponding to a 60.2 ± 3.4% reduction of gene expression in MMP2/AcPEI_15_-transfected PC3. The MMP-2 gene expression was partially restored after 5 days of cell culture in 2D. Consistent with qRT-PCR results, MMP-2 downregulation also results in a significant reduction of the amount of active MMP-2 (*p* < 0.01) detected by enzyme-linked immunosorbent assay (ELISA) in the supernatant of MMP2/AcPEI_15_-transfected PC3 vs. sh/AcPEI_15_-treated group and non-transfected PC3 after 72 h and appears partially recovered after 5 days of treatment (Figure 4B). When MMP2/AcPEI_15_ are dispersed in the collagen matrix (MMP2-3D) transfection efficiency increase over 15 days with a prolonged MMP-2 gene downregulation. Indeed, similar levels of *MMP-2* knockdown were achieved at 5 (58.3%) and 10 days (53.9%) after transfection with MMP2/AcPEI_15_-transfected PC3 compared with sh/AcPEI_15_-treated group and non-transfected PC3 (Figure 4C). A slight increase in *MMP-2* production was seen in MMP2/AcPEI_15_-transfected PC3 (~10%) after 15 days respect to 10- and 5-days treatment. These results were confirmed by ELISA assay (Figure 4D). Western blot analysis confirms gene expression data demonstrating a significant reduction of MMP-2 protein levels in the MMP2-treated group compared with those in the sh/AcPEI_15_-treated group and non-transfected cells (*p* < 0.001) after 3 and 5 days of cell culture in 2D (Figure 4E). This result was confirmed also when MMP2/AcPEI_15_ are dispersed in the collagen matrix (MMP2-3D), where the reduction in MMP2 protein levels (54.6%) is maintained for up to 15 days (*p* < 0.001) as shown in Figure 4F.

### 3.5. MMP2/AcPEI_15_ Inhibition of In Vitro Collagen Degradation Maintain Chondrogenic Markers Expression

**Figure 5:**
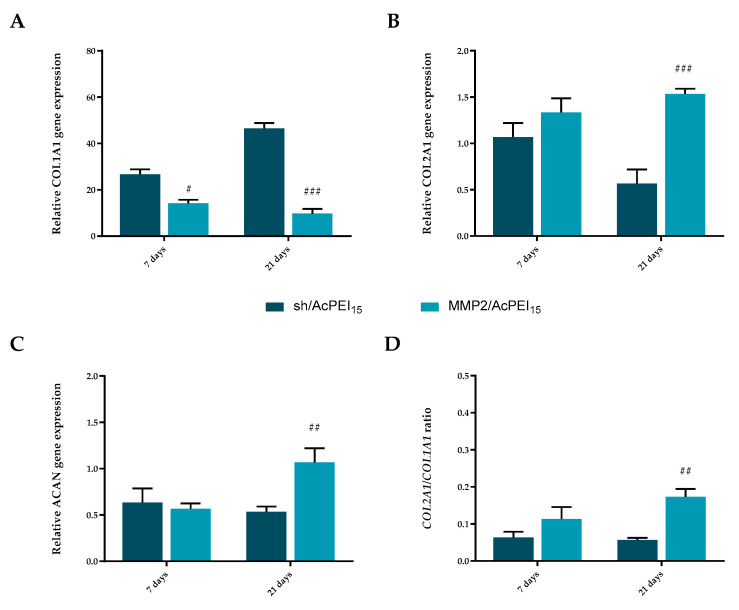
In vitro chondroprotective effect of MMP2/AcPEI_15_. qRT-PCR analysis of (**A**) type I collagen (*COL1A1*), (**B**) type II collagen (*COL2A1*) and (**C**) aggrecan (*ACAN*) in embedded chondrocytes cultured, for 7 and 21 days, presence of conditioned medium derived from MMP2/AcPEI_15_-transfected PC3 cells (MMP2/AcPEI_15_) or conditioned medium from non-transfected cells (sh/AcPEI_15_). The target gene expression was normalized to the housekeeping genes *HPRT1* and *GAPDH*. Relative differences in the PCR results were calculated using the comparative CT (2^−ΔΔCt^) method. The bars represent the means ± standard deviation (n = 3). # *p* < 0.05, ## *p* < 0.01 and ### *p <* 0.001 versus sh/AcPEI_15_. (**D**) The ratios of *COL2A1*/*COL1A1* of MMP2/AcPEI_15_ and of sh/AcPEI_15_ cells were calculated as described in Section 2.

To determine whether MMP2/AcPEI_15_ may exert a protective effect on chondrocyte dedifferentiation induced by collagen matrix degradation and MMP-2 secretion, the differentiation status of the chondrocytes embedded in collagen matrix was investigated by using the qPCR assays after 7 and 21 days (Figure 5). Normal articular cartilage is composed of only one cell type, the chondrocytes, which are normally quiescent and synthesize matrix proteins such as proteoglycans and collagen fibers whose orientation varies throughout cartilage thickness, drawing an “arcade-like” structure [39]. During OA onset, chondrocytes start to release proinflammatory cytokines and express increased amounts of MMPs, leading to inflammation, ECM degradation, and surface fibrillation. In addition, chondrocytes undergo morphological and molecular changes, acquiring the so-called “OA-associated” chondrocytes phenotypes. A de-differentiated-like phenotype is also acquired by a population of OA chondrocytes. It is reflected by (i) the shift from a quiescent to a proliferative state visualized in OA cartilage by cell clustering, (ii) the deposition of fibrosis marker such as type III collagen and α-SMA and (iii) the secretion of the catabolic adipokine leptin whose expression is associated with OA severity [40]. Moreover, this dedifferentiated-like phenotype is characterized by an up and down regulation of specific genes. In particular, it was reported an increased expression of fibroblast marker (type I collagen, *COL1A1*) and a decreased expression of chondrocyte markers (type II collagen *COL2A1* and aggrecan *ACAN*) [41].

It is evident that the expression of chondrocytic de-differentiation related genes *COL2A1* (Figure 5B) and *ACAN* (Figure 5C) were significantly (*p* < 0.001 and 0.01, respectively) increased in cells cultured in the presence of conditioned medium derived from MMP2/AcPEI_15_-transfected PC3 cells (MMP2/AcPEI_15_) compared with conditioned medium of non-transfected cells (sh/AcPEI_15_). Conversely, the absence of MMP-2 knock-down induces a high level of matrix degradation that stimulates chondrocyte de-differentiation, as reflected by a significant up-regulation of *COL1A1* expression in sh/AcPEI_15_ cells compared with MMP2/AcPEI_15_ (Figure 5A). Moreover, the ratio of the mRNA level of *COL2A1* versus that of *COL1A1* (*COL2A1*/ *COL1A1*), which denotes the level of chondrocytic activity, was maintained higher in chondrocytes cultured in the presence of MMP2/AcPEI_15_-transfected PC3 than in those cultured with PC3 alone (Figure 5D). It is worth pointing out that, in this study, we focused on monitoring gene expression rather than the expression of ECM proteins by cells cultured on ECMs because the pre-existing ECM proteins in the culturing substrates interfere with the quantification of newly deposited ECM by chondrocytes.

Together, these results suggest that the suppression of MMP-2 activity prevents the loss of the differentiation phenotype of chondrocytes due to matrix degradation, thus protecting them against degeneration and supporting ECM homeostasis in articular cartilage.

## 4. Conclusions

The cationic AcPEI-NPs were successfully utilized as non-viral vectors to deliver MMP-2 siRNA with the aim of suppressing MMP activity. Such delivery systems are efficiently internalized by cells, and the released siRNA effectively suppresses MMP-2 activity, preventing matrix degradation and supporting ECM homeostasis in articular cartilage. These encouraging results promote further investigation on the described nanocomplex to validate MMP-2 siRNA as ‘‘molecular switch’’ able to support ECM homeostasis and counteract osteoarthritis.

## Figures and Tables

**Table 1:** Primers used for qRT-PCR.

Gene	Forward (5′–3′)	Reverse (5′–3′)
***MMP-2***	CTCAGATCCGTGGTGAGATCT	CTTTGGTTCTCCAGCTTCAGG
***COL1A1***	TGTGCCACTCTGACTGGAAGA	AGACTTTGATGGCATCCAGGTT
***COL2A1***	CTGGTGTGAAGGGTGAGAGT	AGTCCGTCCTCTTTCACCAG
***ACAN***	TCCCCAACAGATGCTTCCAT	GTACTTGTTCCAGCCCTCCT
***ACTB***	ACTCTTCCAGCCTTCCTTCC	CGTACAGGTCTTTGCGGATG

## Data Availability

The data presented in this study are available in the article.

## Supplementary Materials

The following supporting information can be downloaded at: https://www.mdpi.com/article/10.3390/polym15051172/s1, Figure S1. (A) Representative hydrodynamic diameter distribution curves in terms of intensity (%) and (B) zeta potential of MMP2/AcPEIx at different N/P ratios obtained via DLS analysis.
