# Supplementary material for: MMP-2 Silencing through siRNA Loaded Positively-Charged Nanoparticles (AcPEI-NPs) Counteracts Chondrocyte De-Differentiation
Source: Polymers (Basel). 2023 Feb 25;15(5):1172. doi: 10.3390/polym15051172 (PMC10007186; doi:10.3390/polym15051172)
Supplement: Supplementary file 1 [file polymers-15-01172-s001.zip › polymers-2161553-supplementary.pdf]

Article

MMP-2 Silencing through siRNA loaded positively-charged nanoparticles (AcPEI-NPs) counteracts chondrocyte de-differentiation

Raffaele Conte [1#], Mauro Finicelli [3#], Alessia Borrone [2], Sabrina Margarucci [3], Gianfranco Peluso [3,4], Anna Calarco [3,*] and Michela Bosetti [2,*]

1 Elleva Pharma s.r.l. via P. Castellino, 111 – 80131 Naples, Italy
2 Dipartimento di Scienze del Farmaco (DSF), Università Piemonte Orientale "A. Avogadro", Largo Donegani 2, 28100 Novara, Italy
3 Research Institute on Terrestrial Ecosystems (IRET)—CNR, Via Pietro Castellino 111, 80131 Naples, Italy
4 UniCamillus, International Medical University, 00131 Rome, Italy
* Correspondence: anna.calarco@cnr.it (A.C.); michela.bosetti@uniupo.it (M.B.)
\# These authors contributed equally to this work.

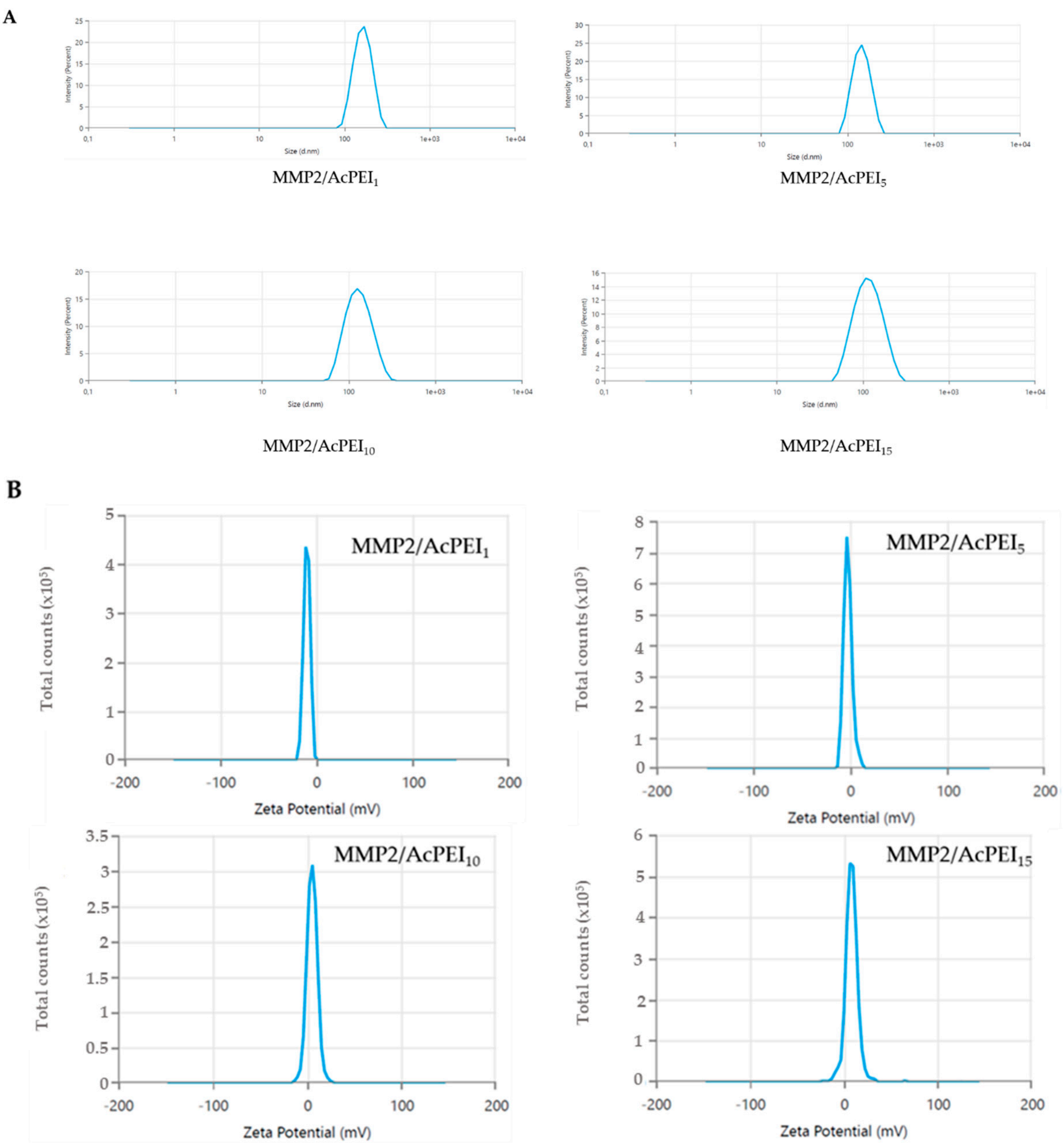

Figure S1. (**A**) Representative hydrodynamic diameter distribution curves in terms of intensity (%) and (**B**) zeta potential of MMP2/AcPEIx at different N/P ratios obtained via DLS analysis (mean ± SD, n = 6).
